# Cerebral autoregulation and neurovascular coupling are progressively impaired during septic shock: an experimental study

**DOI:** 10.1186/s40635-020-00332-0

**Published:** 2020-08-14

**Authors:** Lorenzo Ferlini, Fuhong Su, Jacques Creteur, Fabio Silvio Taccone, Nicolas Gaspard

**Affiliations:** 1 Department of Neurology, Erasme Hospital, Université Libre de Bruxelles, Route de Lennik 808, 1070 Bruxelles, Belgium; 2 Department of Intensive Care, Erasme Hospital, Université Libre de Bruxelles, Bruxelles, Belgium

**Keywords:** Sepsis, Septic shock, Sepsis-associated encephalopathy, Cerebrovascular microcirculation, Neurovascular coupling, Brain dysfunction

## Abstract

**Background:**

Alteration of the mechanisms of cerebral blood flow (CBF) regulation might contribute to the pathophysiology of sepsis-associated encephalopathy (SAE). However, previous clinical studies on dynamic cerebral autoregulation (dCA) in sepsis had several cofounders. Furthermore, little is known on the potential impairment of neurovascular coupling (NVC) in sepsis. The aim of our study was to determine the presence and time course of dCA and NVC alterations in a clinically relevant animal model and their potential impact on the development of SAE.

**Methods:**

Thirty-six anesthetized, mechanically ventilated female sheep were randomized to sham procedures (sham, *n* = 15), sepsis (*n* = 14), or septic shock (*n* = 7). Blood pressure, CBF, and electrocorticography were continuously recorded. Pearson’s correlation coefficient Lxa and transfer function analysis were used to estimate dCA. NVC was assessed by the analysis of CBF variations induced by cortical gamma activity (Eγ) peaks and by the magnitude-squared coherence (MSC) between the spontaneous fluctuations of CBF and Eγ. Cortical function was estimated by the alpha-delta ratio. Wilcoxon signed rank and rank sum tests, Friedman tests, and RMANOVA test were used as appropriate.

**Results:**

Sepsis and sham animals did not differ neither in dCA nor in NVC parameters. A significant impairment of dCA occurred only after septic shock (Lxa, *p* = 0.03, TFA gain *p* = 0.03, phase *p* = 0.01). Similarly, NVC was altered during septic shock, as indicated by a lower MSC in the frequency band 0.03–0.06 Hz (*p* < 0.001). dCA and NVC impairments were associated with cortical dysfunction (reduction in the alpha-delta ratio (*p* = 0.03)).

**Conclusions:**

A progressive loss of dCA and NVC occurs during septic shock and is associated with cortical dysfunction. These findings indicate that the alteration of mechanisms controlling cortical perfusion plays a late role in the pathophysiology of SAE and suggest that alterations of CBF regulation mechanisms in less severe phases of sepsis reported in clinical studies might be due to patients’ comorbidities or other confounders. Furthermore, a mean arterial pressure targeting therapy aiming to optimize dCA might not be sufficient to prevent neuronal dysfunction in sepsis since it would not improve NVC.

## Introduction

Sepsis-associated encephalopathy (SAE) is defined as cerebral dysfunction that accompanies sepsis in the absence of direct central nervous system infection, structural abnormality, or other causes of encephalopathy [1]. Although mostly reversible, SAE is associated with higher short-term mortality [2] and long-term cognitive impairment among survivors [3]. While its physiopathology is not completely understood [1], in addition to a BBB dysfunction [4–6], several studies suggested alterations of cerebral blood flow (CBF) regulation mechanisms as key factors for SAE [7–11]. Post-mortem studies found widespread ischemic lesions in the brain in septic patients, supporting the role of inadequate cerebral perfusion in SAE [12, 13].

Since brain tissue has a high metabolic demand without efficacious system of substrate storage, CBF is strictly controlled to ensure adequate energy supply [14]. CBF is determined by cerebral perfusion pressure (CPP, i.e., the differential between the mean arterial pressure (MAP) and the intracranial pressure), cardiac output, and the vascular tone of the small cerebral vessels [15]. In response to variations of CPP, an adaptation of vascular resistance allows CBF to remain stable, a mechanism known as cerebral autoregulation (CA) [16]. It was originally found that CBF remains constant over a wide range of CPP changes (static cerebral autoregulation, sCA) [16]. Later studies showed that, in that range of CPP values, cerebrovascular autoregulatory capacity depended on the direction (increase vs. decrease) and the speed of changes in CPP [17, 18]. The quantification of fast modifications in cerebrovascular resistance, and consequently in CBF, in relation to rapid changes in MAP within the range of sCA is referred to as “dynamic CA” (dCA). Clinical studies have found alteration of CA in sepsis, especially in the presence of SAE, but they had several confounders: use of vasopressors, variable CO_2_ arterial pressure (PaCO_2_) or temperature, history of hypertension, age, or the presence of extra-cerebral organ dysfunction [8, 9, 19, 20]. Moreover, previous studies included patients with different diseases severity (sepsis and septic shock [21]) evaluated at a variable interval after sepsis diagnosis (between 24 to 72 h) and using different methods (time [8, 9, 20] vs. frequency domain [19]). It is thus still unclear to which extent CA is altered by sepsis itself and which is the temporal course of its disruption.

CBF is also regulated by neuronal activity. This neurovascular coupling (NVC) is responsible for the fine regulation of oxygen and glucose delivery in response to increase in neuronal activity [22]. Whereas previous studies assessed NVC in stimuli-induced conditions both in septic patients and animal models [22, 23], little is known about NVC during spontaneous cerebral activity, which represents most of cerebral activity in mechanically ventilated sedated patients and lends itself more easily to monitoring. Similarly to CA, important confounders, such as age and extra-cerebral organ dysfunction, were not completely accounted for in these studies [22].

The primary aim of this study was thus to determine the time course of alterations of CA and NVC in a clinically relevant animal sepsis model. Our secondary aim was to determine the presence and time course of the associated cortical dysfunction.

## Materials and methods

### General procedure

The Institutional Review Board for Animal Care of the Free University of Brussels (Belgium) approved all experimental procedures (number of Ethical Committee approval: 675 N), which were also in compliance with ARRIVE (Animal Research: Reporting in Vivo Experiments) guidelines. Care and handling of the animals were in accord with National Institutes of Health guidelines (Institute of Laboratory Animal Resources). The protocol was performed on forty female Ovis Aries sheep. We initially planned to allocate animals with a 1:1:1 ratio in the septic (*n* = 16), septic shock (*n* = 16), or sham groups (*n* = 16). For ethical reasons, in order to limit the number of animals, interim analyses were carried out. This allowed reducing the number of animals in the septic shock group to 8. Sample sizes were based on previous studies from our laboratory using the same animal model [7, 24]. After randomization, animals were excluded if they presented a hemoglobin level below 8 g/dl or systemic signs of infection at the moment of the delivery to the laboratory. The general procedures have been described previously [7]; a detailed version is available as Additional Content. Briefly, sheep were mechanically ventilated under general anesthesia provided by continuous IV infusion of ketamine, morphine, and midazolam. Initial doses (ketamine 20 mg kg^−1^ h^−1^; morphine 2 mg kg^−1^ h^−1^; midazolam 3 mg kg^−1^ h^−1^) were adjusted according to electrocorticography (ECOG) in order to achieve a nearly continuous background (i.e., the fraction of ECOG spent in suppression [amplitudes < 10 μV for ≥ 5 s] < 10%). Muscular blockade was achieved using 10 μg.kg^−1^.h^−1^ of rocuronium.

### Surgical procedure

In the sepsis group animals, a midline laparotomy was performed to allow cecum exposure; cecotomy was realized for feces collection (1.5 g.kg^−1^ of body weight), and after local disinfection with iodine solution, the cecum was closed with a double suture and returned to the abdominal cavity. A 25-cm plastic tube (Beldico SA, Marche-En-Famenne, Belgium) was inserted through the laparotomy incision in the abdominal cavity for successive feces injection and secured to the abdominal wall which was successively sutured in two layers. In the sham group, laparotomy was performed in order to provide a systemic post-surgical inflammatory response similar to the sepsis group while avoiding the risk of infection. The animal was then turned in the prone position for the brain surgical procedure. Bilateral craniotomy was performed using a high-speed drill (Wuhu Ruijin Medical instrument, Wuhu, China), and two 2.5 cm^2^ bone holes were opened in the frontal-parietal bones, one on each side, using a laminectomy tool (Aesculap-WerkeAG, Tuttlingen, Germany). The dura mater was opened with scissors, and two 4-contact ECOG electrodes (Dixi Medical, Besançon, France), one per hemisphere, were slipped beneath the dura over the cortex surface of the post-central gyrus and taped to the skull. At a distance of 0.5 cm from the ECOG electrodes, the dura mater was subsequently punctured to insert a laser Doppler flowmetry probe (OxyFlow 4000, Oxford Optronic, UK) for local cerebral blood flow velocity (CBFv) measurement. All catheters were placed under sterile conditions at a depth of 0.5 cm into the brain parenchyma as close as possible one to each other.

### Monitoring and measurements

Ventilator parameters were adjusted to maintain PaO_2_ between 90 and 120 mmHg (12–16 kPa) and PaCO_2_ between 30 and 45 mmHg (4–6 kPa) (as hypercapnia negatively influence CA in sepsis [10]), according to repeated blood gas analysis (Cobas b123, Roche diagnostic, Rotkreuz, Switzerland). Cardiac output and systemic and pulmonary arterial pressure were invasively and continuously measured and recorded simultaneously with ECOG and CBFv with a sampling rate of 250 Hz (Notocord-hem, Instern Company, France). Measurements of mean pulmonary arterial pressure were collected every 1.5 h. Cardiac index (CI) was calculated using standard formulas; the body surface area was estimated from Mitchell’s sheep-specific formula [25].

### Experimental protocol

After the surgical procedures, the animal was allowed to stabilize for 1 h. In all groups, plasmalyte solution and 6% hydroxy-ethyl starch solution (Voluven; Fresenius Kabi, Schelle, Belgium) were titrated to prevent hypovolemia and arterial hypotension.

#### Sepsis vs. sham groups

In the sepsis group, feces were injected into the abdominal cavity, and animals were observed until septic shock (SS) occurred, defined as persisting MAP < 65 mmHg and lactate elevation > 2 mmol/L despite adequate fluid resuscitation [21]; then, animals were sacrificed using IV potassium chloride. From our previous experiences with this model, we saw that after septic shock, the respiratory parameters are difficult to be controlled without a timing vasopressor therapy; since PaCO_2_ highly influences cerebrovascular resistance and dCA assessment, animals in the sepsis group were sacrificed after septic shock. In the sham group, data were collected for 12.5 h; then, animals were sacrificed using IV potassium chloride. All analyses were performed offline, using built-in functions and custom scripts in Matlab (The MathWorks, Natick, MA, USA). Since the interval to develop septic shock differed between septic animals, data from sepsis and sham animals were subdivided into four equal time epochs. The first 30 min for each epoch were selected, and they represented the first four time points (T1_sepsis_–T4_sepsis_); the fifth time point (T5_sepsis_) corresponds to the last 30 min of the whole recording. The same set of analysis was performed for each time point in all animals.

#### Septic shock group

In the septic shock (SS) group, after the initial stabilization period, feces were injected in the abdominal cavity as in the sepsis group. When the response to fluids resuscitation became insufficient, noradrenaline (NA) was started (initial dose 0.5 μg/kg/min) and adjusted to maintain MAP around 80 mmHg. The response to fluid challenge (250 cc infused in 10 min) was deemed insufficient when it resulted in a rise in the CO inferior to 15% of the pre-challenge value [26]. The infusion of NA was maintained and titrated up until the MAP did no longer respond to increase in NA dose and fell below 65 mmHg. Data during NA infusion were subdivided into four time epochs, and four time points (T1_SS_–T4_SS_) were identified by selecting the first 30 min for each epoch; in addition, two 30-min time epochs were selected, one immediately before the onset of the NA infusion (T0_SS_) and the second immediately after NA withdrawal (T5_SS_). Since the end of the recording in the sepsis group slightly differed from the beginning of NA infusion in the septic shock animals, data collected before T0_SS_ were discarded for purpose of rigor.

### Data analysis

#### Data pre-processing

Physiologically implausible values (MAP > 250 mmHg or < 0 mmHg; CBFv > 5000 blood perfusion units (BPU) or < 100 BPU; CO > 15 L/min or < 0 L/min) and artifacts were visually removed, prior to further analysis.

#### Dynamic cerebral autoregulation (dCA)

CBFv signals from the 2 hemispheres were averaged, yielding a mean-CBFv (mCBF). For dCA assessment, 2 linear methods, one in the time domain (Lxa) and the other in the time-frequency domain (transfer function analysis, TFA), were employed using recorded spontaneous fluctuations of MAP and mCBF.

For Lxa, MAP and mCBF were further averaged on 10 s consecutive windows without overlap; then, a Pearson’s correlation coefficient between 30 samples of the averaged values was calculated. Lxa can take any value between − 1 and 1. Values close to 1 indicate linear correlation between variables and thus poor autoregulation whereas values closer to 0, or negative, indicate good autoregulation [27]. In contrast to the previously published Lx index [28, 29], obtained with CPP instead of MAP, Lxa index has never been validated in animal or human studies; as a consequence, no cutoff values are available to define autoregulatory failure. On the other hand, a close association was found between the two laser-flow-Doppler derived methods, Lx and Lxa, and it has been previously shown in our model [7] that CPP variations are superimposable to MAP ones since intracranial pressure do not present notable changes.

We used TFA (Matlab TFA function provided by the Cerebral Autoregulation Research Network [30]) to estimate the gain and the phase shift in the very low frequency range (VLF, 0.02 < Hz < 0.07), where dCA is supposed to be more efficient [31, 32]. Parameters for TFA were window length of 102.4 s, with 59.9% overlap and a Hanning window that led to 41 windows and a spectral resolution of 0.009 Hz. Also, each window contained at least one full period oscillation of the lower frequency considered (0.02 Hz).

#### Neurovascular coupling

**Fig. 1:**
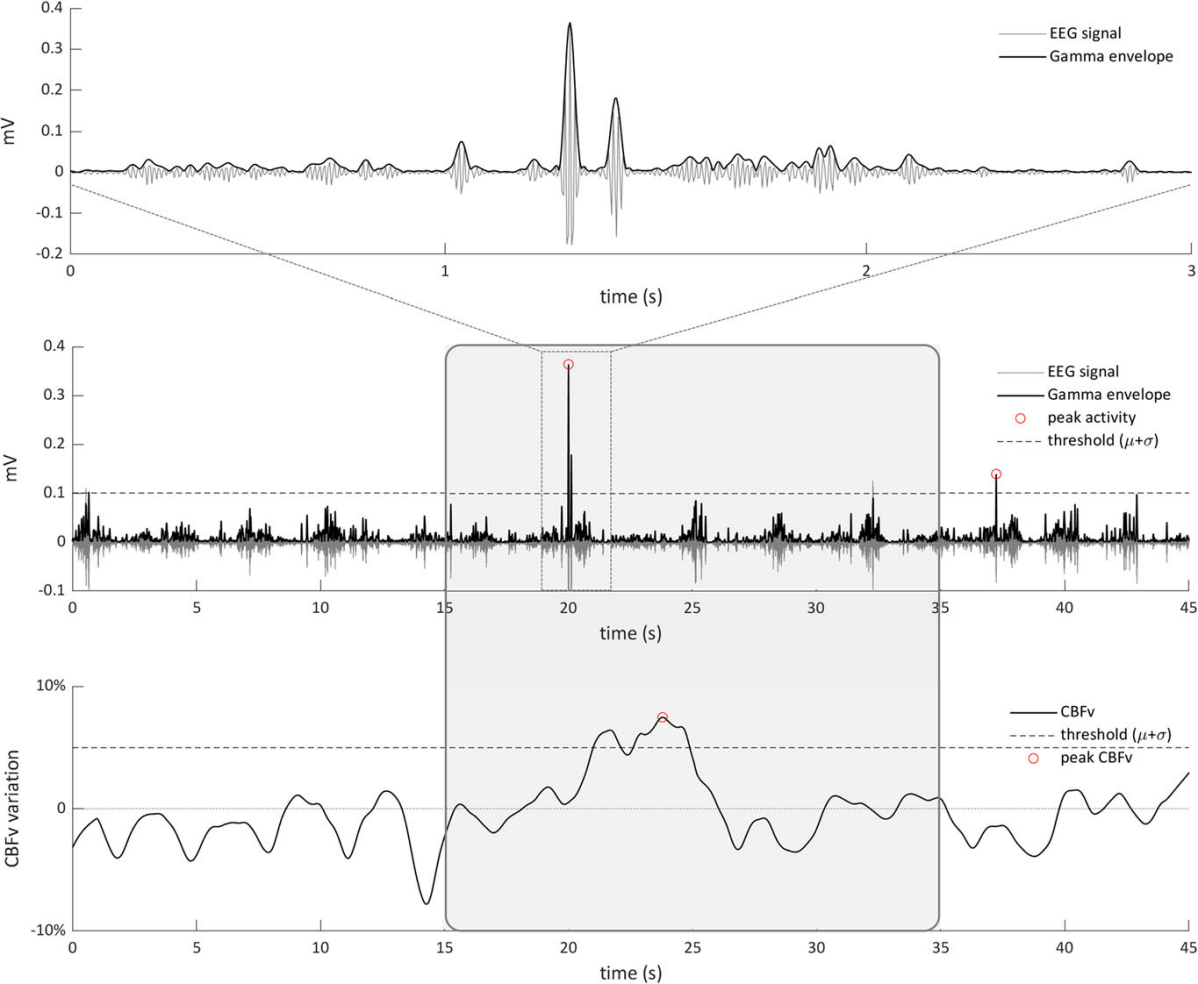
Schematic of analysis techniques used to identify neural events for selecting epochs of the accompanying hemodynamics. Magnification of the gamma filtered EEG signal and the corresponding envelope (Eγ) of a peak of neuronal activity (top row). A 45 s Eγ trial from a single animal is shown as an example (middle row); two peaks of neuronal activity above the chosen threshold are marked. A time epoch of 20 s around the first peak is drawn. Corresponding 45 s of CBFv fluctuation from the same animal (bottom row); an increase in the CBFv appears few seconds after the Eγ peak

The methodological steps are summarized in Fig. 1. CBFv from both hemispheres was filtered using a low-pass zero-phase fourth-order Butterworth filter with a cut-off frequency of 0.25 Hz to limit the higher frequency hemodynamics fluctuations (i.e., due to breathing) [33]. The envelope of the high gamma frequency band (Eγ), a measure of neuronal cortical activity driving NV C[34], was extracted from the ECOG signal using wavelet transform spectral density estimate (*cwt*, *icwt*, and *envelope* functions in Matlab). Neurovascular coupling was subsequently measured using two different approaches. First, NVC driven by spikes of cortical activity was assessed. Briefly, Eγ envelope peaks which exceeded one standard deviation above the mean [35] were detected (*findpeaks* function in Matlab). For each detected Eγ activity peak, CBFv epochs spanning from 5 s before to 15 s after the peak were selected, detrended (*detrend* function in Matlab), and normalized using the mean and standard deviation calculated on the 5 s pre-Eγ peak epoch. If a CBFv peak was identified following the Eγ peak (*findpeaks* function in Matlab), the CBF epoch was included for further analysis. For each included CBFv peak, the normalized amplitude and lag from the corresponding Eγ peak were calculated. We also assessed the spontaneous fluctuations of Eγ (second-level spectrogram) and CBFv with Welch’s periodogram, with an epoch length of 30 min, a window length 180 s, and a window overlap 90%. Previous studies reported that the main peaks of the alpha and theta EEG second-level spectrum were located between 0.01 and 0.02 Hz, between 0.05 and 0.07 Hz, and between 0.1 and 0.25 Hz [36–38]. Since gamma band oscillates with a similar periodicity [39], these frequency bands were used for ECOG second-level spectrum analysis. For CBFv fluctuations, the following frequency subcomponents where used, according to the literature: from 0.02 to 0.04 and from 0.04 to 0.15 [40, 41]. The coherence between Eγ signal and the filtered, detrended and normalized CBFv was then measured by MSC, using the same parameters as for the second-level periodogram. Since the literature does not provide any, we identified peaks in the MSC spectrum of early sham animals (Supplemental Figure S1) and identified their frequency boundaries (0.03 to 0.06 Hz and 0.06 to 0.13 Hz) and used them for further analysis.

#### Alpha-delta ratio

For each time point described above, ECOG spectrograms were calculated using the Welch’s method. The alpha-delta ratio (ADR) was further calculated, as the ratio between power in the alpha (4–8 Hz) and delta (0.5–4 Hz) frequency bands.

### Statistical analyses

Statistical analyses were performed using Matlab (The MathWorks, Natick, MA, USA). A *p* value < 0.05 was considered statistically significant. The sepsis group was compared to the sham group. In the septic shock group, each animal served as its own control; analyses were performed between time points during NA infusion. While NA is known to affect dCA, this effect is lost in case of sepsis [42]. Consequently, it would have been misleading to compare septic animals under NA infusion with sham animals without vasopressors. This also allowed reducing the number of animals.

The Kolmogorov-Smirnov test was performed to assess the normal distribution of values. Data are presented as median and interquartile range (IQR) or median and median absolute deviation. Wilcoxon signed rank test and Friedman test were used to analyze variable differences in time within single groups, Wilcoxon rank sum test, and two-way repeated-measure ANOVA for differences between groups, as appropriate; the linear step-up procedure introduced by Benjamini and Hochberg was applied for controlling the false discovery rate [43]. Tukey-Kramer and Holm post-hoc analysis were employed in case of a corrected *p* value < 0.05.

Since a low MSC could be the result of a noisy signal [44], a cut-off value is necessary to avoid unreliable estimations of gain and phase using the TFA method [45]. The MSC thresholds for a specific frequency were calculated using a Monte Carlo simulation (95% confidence interval based on 100 repetitions of MSC estimation of randomly values adopting standardized parameters recommended in [45] and specified before). In case of non-significant coherence, corresponding gain and phase values were excluded from analysis. Similarly, the coherence between Eγ and CBFv in the assessment of NVC was considered statistically significant if its value for a specific frequency was greater than 95% confidence limit calculated by a Monte Carlo simulation.

## Results

Thirty-six female sheep were included in the protocol (weight, 25 kg [interquartile range (IQR) 22–31]); 4 animals were excluded (Hb < 8 g/dl, *n* = 1 in the septic shock group; systemic signs of infection, *n* = 3, two in the sepsis group and one in the sham group). In sepsis animals (*n* = 14), SS occurred after a median interval of 12.5 h [IQR, 10–13.4 h]; in the sham group (*n* = 15), data were collected for 12.5 h. In septic shock group (*n* = 7), the median interval from the beginning of the experiment to the NA infusion was 14.9 h [IQR, 12–15.1 h]; data were collected for a median of 33.5 h [IQR, 29.7–33.8 h], until spontaneous death occurred.

### Systemic parameters

Evolution of systemic hemodynamics, respiratory, and biological variables over time in the sepsis and the sham animals are presented in Table 1. A significant increase in cardiac index marked the onset of the hyperdynamic state of sepsis and was associated with increase in lactatemia and reduction in the Pa/FiO_2_ ratio.

**Table 1:** Evolution of systemic hemodynamic, respiratory, and biological parameters in sepsis (*n* = 14) and sham (*n* = 15) animals

Variables	Group	Time point 1_**sepsis**_	Time point 2_**sepsis**_	Time point 3_**sepsis**_	Time point 4_**sepsis**_	Time point 5_**sepsis**_	ANOVA group * time (***p***)
**T (°C)**	Sepsis	38.8 (38–40)	39.5 (38.7–40.6)	40.1 (39–40.8)	40.3 (38.6–40.6)	40.1 (38.4–40.4)	0.70
	Sham	40.0 (39.7–40.3)	40.5 (39.9–40.7)	40.7 (40.3–40.8)	40.5 (40.3–40.7)	40.7 (40.2–41)	
**CI (l/min/m2)**	Sepsis	1.8 (1.4–2.3)	2.1 (1.5–2.7)	2.6 (1.9–3.6)	2.7 (2.4–4.0)^*a,b*^	**2.7** (1.8–4.0)^*a,b*^	0.049
	Sham	1.8 (1.6–2.2)	2.2 (2.0–3.0)	2.4 (1.7–2.9)	2.4 (2.0–2.9)	**2.1** (1.6–2.8)	
**MAP (mmHg)**	Sepsis	83 (79–90)	84 (77–90)	83 (75–94)	77 (72–80)	70 (69–72)	0.10
	Sham	97 (94–103)	90 (82–97)	89 (82–89)	84 (82–94)	84 (78–94)	
**MPAP (mmHg)**	Sepsis	10 (5–12)	11 (7–13)	13(12–15)	12 (11–22)	23 (13–25)	0.13
	Sham	8 (5–17)	11 (6–13)	10 (5–13)	11 (6–13)	12 (8–13)	
**PaO**_**2**_**/FiO**_**2**_	Sepsis	332 (293–389)	344 (252–390)	302 (148–358)	185 (110–336)^*a,b*^	**114** (74–253)^*a,b*^	0.034
	Sham	372 (333–385)	365 (328–378)	346 (326–391)	350 (316–389)	**335** (295–367)	
**PaCO**_**2**_ **(mmHg)**	Sepsis	33 (32–36)	33 (33–35)	34 (32–36)	36 (34–38)	36 (34–40)	0.22
	Sham	35 (33–38)	35 (33–37)	34 (33–36)	36 (34–37)	35 (34–36)	
**PaO**_**2**_ **(mmHg)**	Sepsis	114 (105–126)	109 (101–127)	105 (88–116)	89 (75–112)	98 (68–109)	0.14
	Sham	115 (108–126)	114 (109–125)	114 (109–122)	113 (104–121)	113 (104–121)	
**Hb (g/dl)**	Sepsis	8.0 (7.5–9)	9.2 (8.5–9.8)^*a*^	8.6 (8.2–10.3)	8.8 (8.29–9.6)	9.0 (8.0–9.7)	0.09
	Sham	8.5 (7.7–9)	8.7 (7.4–9.4)	8.4 (7.4–9)	8.4 (7.1–8.7)	8.2 (7.6–9.0)	
**Lactate (mmol/l)**	Sepsis	0.5 (0–1.2)	0.6 (0.0–1.4)	**1.2** (0–1.8)	**1.3** (1.1–1.9)^*a,b*^	**1.9** (1.1–2.4)^*a,b*^	0.002
	Sham	0.0 (0–1)	0.5 (0.0–1.1)	**0.0** (0–0)	**0.0** (0–0)	**0.0** (0–0)	
**pH**	Sepsis	7.43 (7.38–4.47)	7.45 (7.39–7.47)	7.42 (7.37–7.45)	7.40 (7.32–7.41)	7.33 (7.29–7.39)	0.14
	Sham	7.45 (7.42–7.48)	7.44 (7.40–7.46)	7.41 (7.35–7.43)	7.40 (7.34–7.41)	7.40 (7.33–7.41)	
**Urine output (ml)**	Sepsis	0 (0–0)	250 (125–345)	459 (200–560)	721 (300–890)	691 (443–1091)	0.06
	Sham	0 (0–0)	250 (141–506)	573 (408–973)	935 (593–1468)	1180 (858–1740)	
**Fluids amount (ml)**	Sepsis	800 (573–952)	1666 (888–2329)	2474 (1580–2889)	3400 (2045–4529)	4558 (2300–5616)	0.06
	Sham	659 (500–1075)	1272 (820–1732)	1935 (1498–2292)	2915 (1909–3119)	3090 (1832–3434)	
**Midazolam (mg/kg)**	Sepsis	11 (10–11)	14 (13–15)	16 (15–17)	17 (17–20)	**19** (18–23)	0.029
	Sham	11 (10–11)	16 (13–17)	19 (17–23)	22 (20–26)	**25** (22–31)	
**Ketamine (mg/kg)**	Sepsis	70 (64–70)	93 (89–99)	104 (103–116)	116 (112–134)	**126** (117–150)	0.029
	Sham	70 (64–70)	104 (89–116)	125 (110–150)	146 (131–176)	**168** (144–204)	

Data are presented as median and (IQR). In case of significant ANOVA group-time interaction, post-hoc analysis is presented. Significant at 5% vs. (^*a*^) time point 1 or (^*b*^) time point 2 in the within-group analysis. Significant differences between groups at a specific time point are bold

*°C* Celsius degrees, *CI* cardiac index, *MAP* mean arterial pressure, *MPAP* mean pulmonary arterial pressure, *PaO*_*2*_*/FiO*_*2*_ ratio used to identify and quantify acute lung injury (acute lung injury if ratio ≤ 300, acute respiratory distress syndrome if ratio ≤ 200)

Table 2 shows the evolution of the same systemic parameters during the septic shock phase. NA infusion allowed to maintain a value of MAP around 80 mmHg (median 78 mmHg; IQR, 74.5–80.4); NA total doses were similar between animals (5.1 ± 0.3 mg/kg). PaCO_2_ and PaO_2_ did not vary significantly throughout the experiment. A slight and non-significant increase in PaCO_2_ was observed in the late time points (T_4SS_) in the septic shock animals (Table 2). Nevertheless, no animal had PaCO_2_ values outside the normal range.

**Table 2:** Evolution of systemic hemodynamics, respiratory, and biological variables over time in septic shock (*n* = 7) animals

Variables	Time point 0_**SS**_	Time point 1_**SS**_	Time point 2_**SS**_	Time point 3_**SS**_	Time point 4_**SS**_	Time point 5_**SS**_	Friedman (***p***)
**Noradrenaline**	Off	On	On	On	On	Off	NA
**T (°C)**	39.3 (38.1–40.3)	39.3 (38.7–40.3)	40.0 (39.6–40.7)	40.7 (40.7–40.8)	40.6 (40.1–40.8)	40.0 (39.7–41.0)	< 0.001
**CI (l/min/m2)**	1.7 (1.5–2.3)	2.2. (1.8–2.4)	2.2 (2.2–4.0)	2.3 (2.3–3.7)	1.4 (1.0–2.6)	1.1 (0.7–2.4)	0.11
**MAP (mmHg)**	78 (72–80)	79 (75–81)	78 (77–83)	78 (77–80)	73 (71–79)	51 (48–55)^*b,c,d*^	**0.009**
**MPAP (mmHg)**	15 (11–17)	19 (17–23)	26 (21–29)	29 (24–33)	29 (26–32)^*b*^	30 (26–35)	**< 0.001**
**PaO**_**2**_**/FiO**_**2**_	383 (327–399)	340 (310–378)	288 (226–302)	235 (175–292)	175 (159–223)	166 (135–199)	0.05
**PaCO**_**2**_ **(mmHg)**	35 (34–35)	35 (33–38)	37 (34–43)	37 (36–40)	39 (38–41)	38 (30–39)	0.05
**PaO**_**2**_ **(mmHg)**	114 (108–123)	104 (98–113)	91 (83–97)	105 (97–112)	106 (98–122)	104 (97–120)	0.31
**Hb (g/dl)**	8.7 (7.7–9.6)	9.7 (7.2–11.2)	9.9 (8–10.4)	9.6 (8.2–10.1)	7.4 (6.2–9.1)	5.5 (4.9–7.8)	0.003
**Lactate (mmol/l)**	1.4 (0.9–1.8)	1.6 (1.0–2.4)	2.3 (1.9–2.7)	4.8 (3.6–5.8)	6.4 (6–10)	8.7 (8.7–10.3)^*a,b,c,d*^	**< 0.001**
**pH**	7.38 (7.37–7.46)	7.36 (7.34–7.38)	7.26 (7.23–7.33)	7.12 (7.04–7.28)	6.9 (6.81–7.17)	6.9 (6.77–7.02)^*c,d*^	**< 0.001**
**Midazolam(mg/kg)**	29 (25–40)	36 (30–43)	44 (43–49)	57 (53–59)	59 (57–68)	60 (57–70)	**< 0.001**
**Ketamine (mg/kg)**	194 (164–265)	237 (201–290)	296 (284–329)	378 (352–395)	396 (383–453)	401 (383–467)	**< 0.001**

Data are presented as median and (IQR). Only significant post-hoc analysis is presented; the corresponding ANOVA *p* values are bold. Significant at 5% vs (^*a*^) time point 0_SS_, (^*b*^) time point 1_SS_, (^*c*^) time point 2_SS_, and (^d^) time point 3_SS_ in the within-group analysis

*°C* Celsius degrees, *CI* cardiac index, *MAP* mean arterial pressure, *MPAP* mean pulmonary arterial pressure, *PaO*_*2*_*/FiO*_*2*_ ratio used to identify and quantify acute lung injury (acute lung injury if ratio ≤ 300, acute respiratory distress syndrome if ratio ≤ 200), *Hb* hemoglobin, *SS* septic shock

### Cerebral autoregulation

**Fig. 2:**
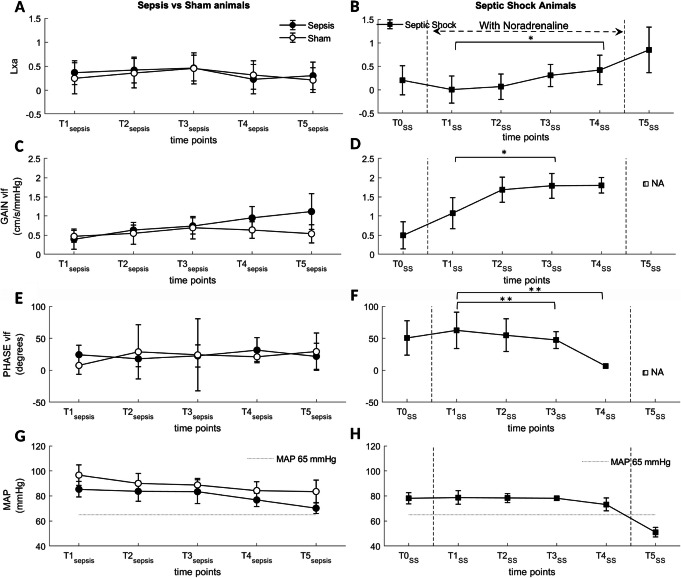
Temporal evolution of dynamic cerebral autoregulation parameters during experimental sepsis. Lxa (**a**, **b**), TFA gain (**c**, **d**), and phase (**e**, **f**), and of mean arterial pressure (**g**, **h**) in sepsis vs. sham (**a**, **c**, **e**, **g**) and septic shock (**b**, **d**, **f**, **h**). No difference was observed between the sepsis and sham groups but a progressive increase in Lxa, TFA gain, and a progressive decrease in TFA phase was observed in the septic shock group, indicating progressive loss of dCA. N/A = not available data, due to coherence values below significance (see the “Materials and Methods” section). Data are presented as median ± mean absolute deviation. *p < 0.05; **p < 0.01

Changes in the dCA parameters at different time points are summarized in Fig. 2. We found no difference in the Lxa or in the TFA parameters between the sepsis and sham groups. In the septic shock animals, the Lxa significantly increased between T1_SS_ and T4_SS_. Upon NA withdrawal, Lxa approached unity, indicating complete loss of dCA. Both TFA parameters showed comparable trends towards a late reduction in the efficacy of dCA in the septic shock group, approximately 5 ± 1.8 h before Lxa. We found no significant correlations between dCA parameters (TFA and Lxa, *p* = 0.19 and *p* = 0.46, respectively) and PaCO_2_ (*p* = 0.27).

### Neurovascular coupling

**Fig. 3:**
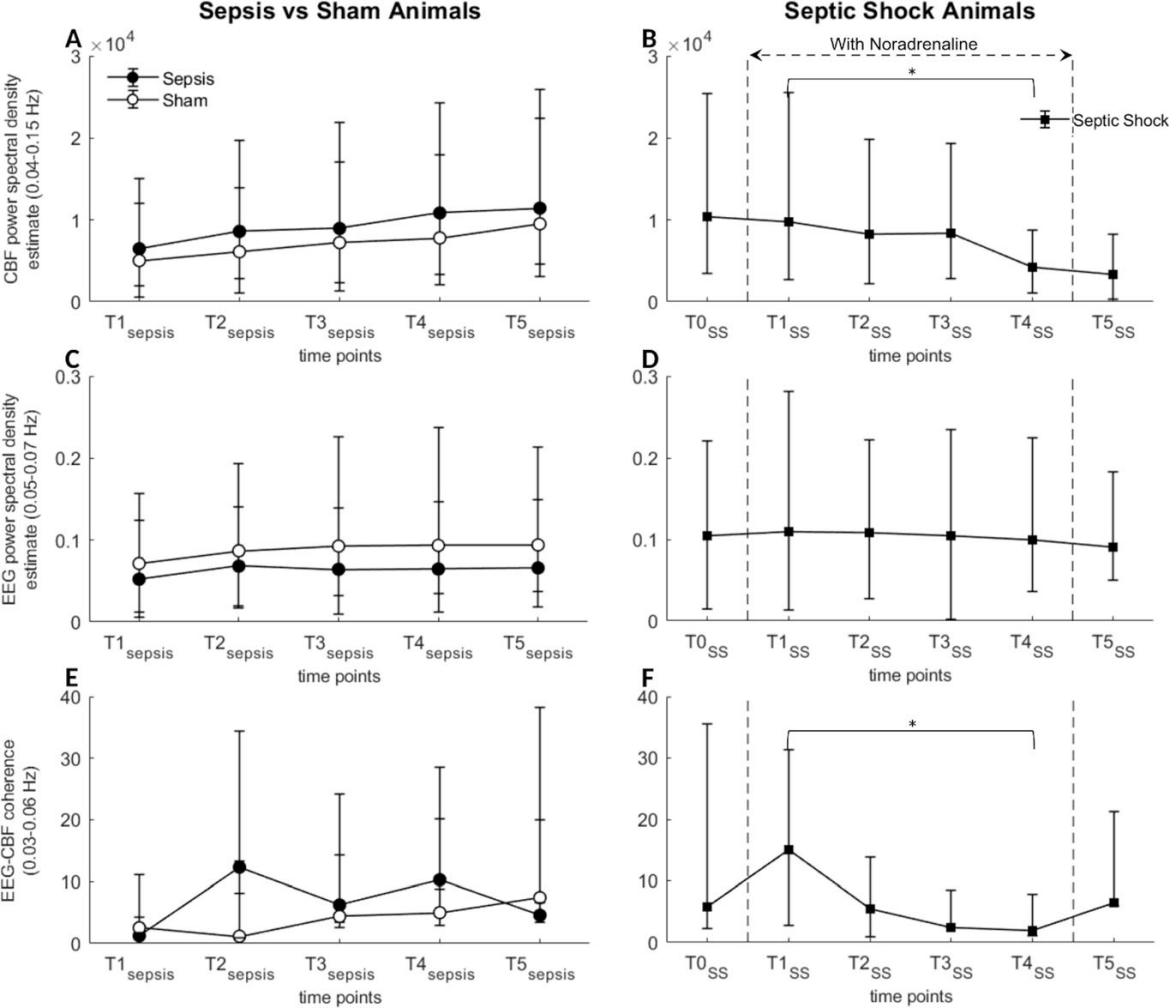
Temporal evolution of neurovascular coupling measured by coherence between periodic fluctuations of cortical activity and cerebral blood flow. Cerebral blood flow velocity (CBFv) spectral power (0.04–0.15 Hz frequency band; **a**, **b**), second-level spectral power of gamma activity envelope (Eγ; 0.05–0.07 Hz frequency band; **c**, **d**), and their magnitude-squared coherence (MSC; 0.03–0.06 Hz frequency band; **e**, **f**) in sepsis vs. sham groups (**a**, **c**, **e**) and in septic shock group (**b**, **d**, **f**), expressed as median and inter-quartile range. *p < 0.05 by RMANOVA test analysis. No significant difference, neither in CBFv nor in Eγ spectra fluctuations nor in MSC, was observed in sepsis and sham animals in any of the frequency bands considered. In the septic shock group, there was a significant decrease between T1_SS_ and T4_SS_ in the spectrum of CBFv fluctuations between 0.04 and 0.15 Hz and a significant decrease in MSC for frequencies between 0.03 and 0.06 Hz. Comparative analysis for other frequencies of CBFv and Eγ spectral power and MSC are presented in Supplemental Figure S3 (a, b, c). Power spectral density estimation are expressed in √dB/Hz

We found no significant difference in the percentage of Eγ peaks followed by CBFv peaks, the amplitude and lag of the CBFv peaks, between or within the sham and sepsis groups, although there was a trend towards a decrease in Eγ peaks followed by CBFv peaks in the last time point in the sepsis group (Supplemental Figure S2). Similarly, none of the parameter showed a significant change during septic shock, although there was again a decreasing trend in the percentage of Eγ peaks followed by CBFv peaks with progressing sepsis. The second-level Eγ spectrum, the periodogram of CBFv, and the MSC between Eγ and CBF did not show any significant change in the sham and sepsis groups (Supplemental Figure S3a (A,C), S3b (A,C,E), S3c (A,C)). However, we observed a significant decrease between T1_SS_ (immediately after NA) and T4_SS_ (last time point prior to cessation of NA) in spontaneous fluctuations of CBFv in frequencies between 0.04 and 0.15, as well as in MSC between Eγ and CBFv in frequencies between 0.03 and 0.06 Hz (Fig. 3).

### Cortical activity

**Fig. 4:**
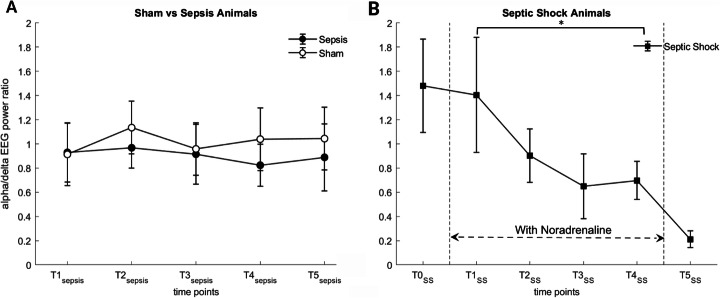
Temporal evolution of cortical activity during experimental sepsis. Alpha/delta ratio of the ECOG signal in sepsis vs. sham (**a**) and septic shock (**b**) group. No difference is observed in the sepsis and sham groups. There is a significant decrease in ADR in the septic shock group between T1_SS_ and T4_SS_. Data are presented as median ± mean absolute deviation; *p < 0.05

Finally, as shown in Fig. 4, the alpha/delta ratio of the ECOG signal remained stable during sepsis but showed a significant decrease during septic shock.

## Discussion

We formally demonstrate, in a clinically relevant animal model, that dCA, NVC, and cortical functions are impaired at the time of septic shock, supporting the hypothesis that SAE is, at least in part, a consequence of relative cerebral hypoperfusion.

Animal studies prior to this one concluded that CA was not altered in sepsis but only measured static CA (sCA) [46–48], a finding that was in line with human studies [11, 19, 49]. Our findings are thus more in line with clinical studies that suggested that dCA is altered during sepsis [8, 9, 19, 20], particularly [8, 9] or exclusively [20] in patients with SAE. Of note, a dissociation of sCA and dCA has been also observed in other clinical settings, such in anesthetized or in stroke patients [50, 51]. Our model also minimizes chronic and acute confounders that were unavoidable in human studies and allows us to conclude that mechanisms of dCA are impaired per se at the time of septic shock. The longitudinal analysis indicates that dCA impairment is a dynamic process, as suggested by prior experiments with LPS infusion [19, 52, 53]. Both time-domain (Lxa) and frequency-domain (TFA) metrics yielded similar results, but TFA showed changes earlier than Lxa. Although experimental studies have shown a poor sensitivity of gain alone as indicator of autoregulatory performance, the concomitant reduction in the phase shift, associated with a significant coherence, makes this finding more reliable [54]. Nevertheless, no previous studies have compared Lxa and TFA performance in describing variations in autoregulation, and this question is beyond the scope of this work. We thus cannot draw any firm conclusion on the comparative sensitivity and specificity of both approaches.

As seemingly opposed to some human studies, however, the alteration in dCA occurred only after the onset of septic shock in our model. Again, we made this observation at controlled normal MAP levels in otherwise healthy animals, which strongly indicates an intrinsic alteration of dCA. Although we did not formally demonstrate it, this acute alteration probably indicates a narrowing of the plateau of the autoregulatory curve, rather than a shift in the overall curve [55]. On the other hand, clinical studies have shown that the ranges of the plateau of the autoregulatory curve are highly variable between individuals [56, 57], with elderly patients and those with a chronic hypertension demonstrating a right shift of the curve, i.e., elevated lower limit of the plateau [58, 59]. As age and pre-existing cerebrovascular disorders are also well-known risk factors of SAE [60–62], it is thus possible that the alteration of CA in the less severe phases of sepsis observed in some studies [9, 20, 63] is due to a level of MAP below the lower limit of a right-shifted autoregulatory curve, rather than an alteration of CA per se. Of note, although LPS infusion does not fully recapitulate the pathophysiology of sepsis, an experimental study in healthy volunteers receiving LPS, which mimics the early phase of sepsis, did not demonstrate an alteration of CA [64]. It is also possible that patients with chronic cerebrovascular disorder might show earlier sensitivity to sepsis-induced alterations in dCA.

Whereas earlier studies assessed NVC in stimuli-induced conditions both in septic patients and animals models [22, 23], little is known about NVC during spontaneous brain activity. In contrast to these studies, which found a reduction in amplitude [22] and a delay [23] in the CBFv response to stimulus-induced cortical activity, we found no significant variations in the amplitude or in the time lag of the CBFv response, independently from the EEG frequency band assessed, even if a trend in the reduction of the CBFv response was observed when comparing the percentage of EEG peaks followed by a CBFv peak. These differences with previous studies could be due to the fact that the magnitude of spontaneous variations in cortical activity is not sufficient to challenge CBF regulation. However, an alteration in the coherence between cortical activity and CBFv was observed during septic shock, indicating a reduced efficiency of cerebral vessels to adjust CBF to spontaneous fluctuations of cortical activity.

Finally, alterations in cortical activity, as assessed by the ADR, also became evident at the time dCA and NVC were altered, after the onset of shock, and despite a MAP maintained within a normal range. This temporal sequence thus suggests that the pathophysiological mechanisms of CA and NVC impairment might be similar to those leading to systemic circulatory failure, such as general microvascular dysfunction [19]. These findings also indicate that the intrinsic alterations in the mechanisms of CBF homeostasis are indeed causing part of the cortical dysfunction associated with sepsis. Most studies on carbon dioxide-induced cerebral vasoreactivity in sepsis also confirm cerebral microcirculation failure [10, 65]. Other animal studies showed structural microcirculation changes induced by sepsis, such as the reduction in the proportion of cortical perfused vessels [7] probably attributable to endothelial adhesion of leucocytes and platelet [66], astrocyte end-feet [5], and pericytes [67] detachment from the vessel walls, and increased blood-brain barrier (BBB) permeability [4, 68] contributing to altered extracellular milieu of the brain and to disrupt the neurovascular unit. Previous studies suggested a prominent role of BBB dysfunction, mediated by cytokines such as TNF-α in SAE [4, 6, 69]. In humans, severe encephalopathy assessed by EEG was associated with higher CSF protein level of protein, suggestive of an increase in BBB permeability [70]. These structural changes at the endothelial and blood-brain barrier levels may be partly responsible for the functional cerebrovascular dysfunction we described. Furthermore, endothelin-1 contributes to CBF impairment by stimulating brain inflammation in sepsis [71]. Endothelin receptors antagonist have shown some promising results on microcirculation and mitochondrial dysfunction in sepsis [72, 73], and their effect on dCA and NVC could be further studied in our model.

Our study provides some leads for future clinical applications and studies. First, with the limitations discussed above, our results suggest that it could be possible to replace stimulus-induced assessment of NVC by analysis of the coherence of spontaneous fluctuation of CBF and neuronal activity. Since NVC reflects a physiologically complemental aspect of CBF regulation, it should be assessed as frequently as cerebral autoregulation, but the lack of standardized and automated protocol of analysis for critically ill patients limits the utility of stimulus-induced NVC assessment in clinical practice [74]. Further studies in humans are, therefore, necessary to evaluate feasibility and efficacy of non-stimulus-induced NVC assessment. Second, the observation that not only CA but also NCV is impaired during septic shock also raises the possibility that strategies aiming at optimizing MAP or even CA might not fully prevent the brain from injuries caused by loss of NVC. Structural changes potentially responsible for the neurovascular unit disruption, as discussed above, are not reversed by optimization of CPP. Further research should focus on strategies preventing or reversing neurovascular unit disruption (for example, reducing oxidative stress [74]) in order to preserve NVC function. Again, it would be required to assess NVC in the ICU. Finally, the integration of dCA and NVC concepts into prognostic and therapeutic approaches would improve the clinical application of these tools. This may guide future randomized trials in choosing the right therapeutic intervention (optimal target MAP therapies in patients with dCA alteration only and coupled therapies in those presenting NVC impairment too) in order to show if focusing on cerebral circulation improves care of septic patients, as it seemed to be the case in preliminary studies in other conditions [75, 76].

Our study presents some limitations. The laser Doppler technique has been widely employed to measure regional changes in CBF thanks to its high temporal resolution [23, 77]. However, this technique presents several limitations, not least the fact that it measures velocity rather than flow, it overestimate high increase in CBF in comparison with some reference methods [78], and absolute values are extremely heterogenous [79]; for this reason, results have to be interpreted cautiously. We followed some procedures to circumvent these limitations. The probe was secured in its initial position in order to prevent artifacts. Moreover, for each animal and for each time point, CBFv data were normalized and averaged across the two hemispheres, before calculation of Lx and TFA, both measures reflecting the relation between CBF and MAP fluctuations rather than absolute values. Second, it is well known that the pressure-flow relationship is nonlinear [31, 55] because of the variation in vascular resistance as a consequence of changes in MAP and the effect of other parameters, such as PaCO_2_, that might influence CBF. Since the complexity of most nonlinear methods precludes the physiological interpretation of the results [55], linear models are usually employed, and a lack of linearity between MAP and CBF in these models is interpreted as a normal CA [45]. A lack of linearity could also be the result of a low signal-to-noise ratio leading to a misinterpretation of data. In order to circumvent this issue, we estimated minimal meaningful coherence thresholds and excluded gain and phase unreliable results. Thus, our results should be robust and reliable. Moreover, there were no significant correlations between dCA parameters and PaCO_2_, ruling out the possibility that PaCO_2_ variations could have confounded dCA estimations. Third, previous studies examined the influence of sedation on dCA, showing that it is enhanced by midazolam and probably also by ketamine [80, 81], both of which were used in these experiments. However, although we cannot rule out the possibility that absolute dCA values might have been influenced by sedation, especially ketamine, we found no statistically significant differences between the sepsis and sham groups. Further, prolonged experiments, associated with an increasing cumulative amount of sedative drugs, disclosed a decrease in dCA. The effect of sedation on NVC at the moment is unclear. Fourth, high doses of NA can affect dCA [42]. A study in healthy volunteers has suggested that NA effect on cerebral vasculature might be partially due to increase in ventilation via a β-adrenergic stimulation of the carotid chemoreceptors [42]. Since animals were mechanically ventilated, NA could not influence ventilation frequency. So, as septic shock animals served as their own control and NA-induced PaCO_2_ variations were controlled by mechanical ventilation, the NA influence on CA assessment was probably limited. Finally, in order to limit for potential confounders, we did not employ specific sepsis therapies, such as antibiotics, since it has been shown that they can modify BBB [82], influence central nervous system inflammation [83, 84], and induce seizures [85]; on the other hand, this do not allow an entire translation of our results to clinical practice. Furthermore, albeit sheep is a promising surrogate for modelling human brain diseases [86], interspecies differences limit the generalization of animals studies results to humans.

## Conclusions

In this clinically relevant model of sepsis, we observed a progressive loss of dCA and NVC, in septic shock, and this was associated with cortical neuronal dysfunction. These findings indicate that the alteration of mechanisms controlling cortical perfusion plays a role in the pathophysiology of SAE; both dCA and NVC should be assessed in clinical practice and therapies targeting only CA could not be efficacious to prevent neuronal dysfunction. NVC assessment without stimulation protocols could increase the employment of this tool in clinical practice. Further study should aim to clarify the molecular mechanisms of dCA and NVC alterations and to develop prognostic and therapeutic approaches as well.

## Supplementary information

**Additional file 1.** This section contains five additional figures and a word file with detailed Materials and Methods section and figure legends. Supplemental figure S1 provides a representation of power spectra of magnitude-squared coherence in sham group used in the neurovascular coupling analysis process, while supplemental figure S2 and S3 (a,b,c) provide additional results pertaining to neurovascular coupling analysis.

## Data Availability

The datasets used and/or analyzed during the current study are available from the corresponding author on reasonable request.

## Abbreviations

ADR: Alpha-delta ratio
BBB: Blood-brain barrier
BPU: Blood perfusion unit
CA: Cerebral autoregulation
sCA: Static cerebral autoregulation
dCA: Dynamic cerebral autoregulation
CBF: Cerebral blood flow
mCBF: Mean cerebral blood flow
CBFv: Cerebral blood flow velocity
CI: Cardiac index
CO: Cardiac output
CPP: Cerebral perfusion pressure
ECOG: Electrocorticography
EEG: Electroencephalogram
Eγ: Envelope of the cortical high gamma activity
HR: Heart rate
IQR: Interquartile range
MAP: Mean arterial pressure
MPAP: Mean pulmonary arterial pressure
MSC: Magnitude-squared coherence
NA: Noradrenaline
NVC: Neurovascular coupling
PaCO_2_: Arterial carbon dioxide partial pressure
PaO_2_: Arterial oxygen partial pressure
PEEP: Positive end-expiratory pressure
SAE: Sepsis-associated encephalopathy
SS: Septic shock
TFA: Transfer function analysis
VLF: Very low frequencies
