# Supplementary material for: Cerebral autoregulation and neurovascular coupling are progressively impaired during septic shock: an experimental study
Source: Intensive Care Med Exp. 2020 Aug 14;8:44. doi: 10.1186/s40635-020-00332-0 (PMC7426896; doi:10.1186/s40635-020-00332-0)
Supplement: Supplementary file 1 — Additional file 1. This section contains five additional figures and a word file with detailed Materials and Methods section and figure legends. Supplemental figure S1 provides a representation of power spectra of magnitude-squared coherence in sham group used in the neurovascular coupling analysis process, while supplemental figure S2 and S3 (a,b,c) provide additional results pertaining to neurovascular coupling analysis. [file 40635_2020_332_MOESM1_ESM.zip › Ferlini_Supplemental material.docx]

**ADDITIONAL CONTENT**

**Cerebral autoregulation and neurovascular coupling are progressively impaired during septic shock: an experimental study**

*Lorenzo Ferlini, MD^1^; Fuhong Su, MD, PhD^2^; Jacques Creteur, MD, PhD^2^; Fabio Silvio Taccone, MD, PhD^2^; Nicolas Gaspard, MD, PhD^1^*

Affiliations

^1^Department of Neurology, Erasme Hospital, Université Libre de Bruxelles, Bruxelles, Belgium.

^2^Department of Intensive Care, Erasme Hospital, Université Libre de Bruxelles, Bruxelles, Belgium.

**Additional content:**

1. Detailed MATHERIAL and METHODS section + bibliography
2. Supplemental figure legend

**MATERIALS AND METHODS**

***General procedure.***

The Institutional Review Board for Animal Care of the Free University of Brussels (Belgium) approved all experimental procedures (number of Ethical Committee approval: 675N), which were also in compliance with ARRIVE (Animal Research: Reporting in Vivo Experiments) guidelines. Care and handling of the animals were in accord with National Institutes of Health guidelines (Institute of Laboratory Animal Resources). The protocol was performed on forty female Ovis Aries sheep. We initially planned to randomly allocated animals with a 1:1:1 ratio in the septic (n = 16), septic shock (n=16) or sham groups (n = 16). For ethical reasons, in order to limit the number of animals, intermediate analysis were carried out. This allowed reducing the number of animals in the septic shock group to 8. Sample sizes were based on previous studies from our laboratory using the same animal model[1, 2]. After randomization, animals were excluded if they presented a hemoglobin level below 8 g/dL or systemic signs of infection at the moment of the delivery to the laboratory. The animal was fasted for 12 h prior to the experiment (single 2.4m^2^ cage, room temperature 20-22° C with standard light/dark cycle) with free access to water. On the morning day of the experiment, intramuscular injection of midazolam (0.25 mg/kg—Dormicum; Roche SA, Beersel, Belgium) and ketamine hydrochloride (20 mg/kg—Imalgine; Merial, Lyon, France) was used as premedication. After intravenous injection of fentanyl (30 μg/kg—Fentanyl; Janssen Pharmaceutica, Beersel, Belgium) and rocuronium (0.1 mg/kg—Esmeron; Organon, Oss, the Netherlands), tracheal intubation was performed (Tracheal Tube, 8.0; Hi-Contour, Mallinckrodt Medical, Athlone, Ireland). Volume-controlled mechanical ventilation (Servo ventilator 900 C; Siemens-Elema, Solna, Sweden) was used with standardized parameters: tidal volume of 10 mL/kg, respiratory rate of 12–16 breaths/min, positive end-expiratory pressure of 5 cm H2O, FiO_2_ of 0.3, and inspiratory time/expiratory time of 1:2. A continuous IV infusion of ketamine, morphine, and midazolam was used as general anesthesia throughout the entire experiment as in [1]. Initial doses (ketamine 20 mg·kg^-1^·h^-1^; morphine 2 mg·kg^-1^·h^-1^; midazolam 3 mg·kg^-1^·h^-1^) were adjusted according to electrocortigraphy (ECOG) in order to achieve a nearly continuous background (i.e. the fraction of ECOG spent in suppression [amplitudes < 10 µV for ≥ 5 s] < 10%). Muscular blockade was achieved using 10 μg.kg^-1^.h^-1^ of rocuronium. A Foley catheter (14F; Beiersdorf AG, Hamburg, Germany) was placed in the bladder for continuous urine output monitoring.

***Surgical Procedure.***

An arterial catheter (4.5F Vygon, Cirencester, UK) was placed in the right carotid artery after surgical vessel exposure. A 7F pulmonary artery catheter (Edwards Life Sciences, Irvine, CA) was advanced into a pulmonary artery, under monitoring of pressure wave-forms, via an introducer placed in the right external jugular vein. The catheters were connected to pressure transducers (Edwards Life Sciences). In the sepsis group animals, a midline laparotomy was performed to allow cecum exposure; cecotomy was realized for feces collection (1.5 g.kg^-1^ of body weight) and, after local disinfection with iodine solution, the cecum was closed with a double suture and returned to the abdominal cavity. A 25-cm plastic tube (Beldico SA, Marche-En-Famenne, Belgium) was inserted through the laparotomy incision in the abdominal cavity for successive feces injection and secured to the abdominal wall which was successively sutured in two layers. In the sham group, laparotomy was performed in order to provide a systemic post-surgical inflammatory response similar to the sepsis group while avoiding the risk of infection. The animal was then turned in the prone position for the brain surgical procedure. Bilateral craniotomy was performed using a high-speed drill (Wuhu Ruijin Medical instrument, Wuhu, China) and two 2.5 cm^2^ bone holes were opened in the frontal-parietal bones, one on each side, using a laminectomy tool (Aesculap-WerkeAG, Tuttlingen, Germany). The dura mater was opened with scissors and two 4-contact ECOG electrodes (Dixi Medical, Besançon, France), one per hemisphere, were slipped beneath the dura over the cortex surface of the post-central gyrus and taped to the skull. At a distance of 0.5 cm from the ECOG electrodes, the dura mater was subsequently punctured to insert a laser-Doppler flowmetry probe (OxyFlow 4000, Oxford Optronic, UK) for local cerebral blood flow velocity (CBFv) measurement. All catheters were placed under sterile conditions at a depth of 0.5 cm into the brain parenchyma as close as possible one to each other.

***Monitoring and Measurements.***

Ventilator parameters were adjusted to maintain PaO_2_ between 90 and 120 mmHg (12-16 kPa) and PaCO_2_ between 30 and 45 mmHg (4-6 kPa) (as mild hypercapnia negatively influence CA in sepsis[3]), according to repeated blood gas analysis (Cobas b123, Roche diagnostic, Rotkreuz, Switzerland). Mean arterial pressure (MAP), heart rate (HR), mean pulmonary arterial pressure (MPAP) (SC 9000 monitor; Siemens, Berlin, Germany), core-temperature and cardiac output (CO) (Vigilance II monitor; Edwards Lifesciences, Irvine, California, United-States) were monitored continuously. Systemic hemodynamic parameters, ECOG, and CBFv were recorded continuously and simultaneously with a sampling rate of 250Hz (Notocord-hem, Instern Company, France). Measurements of mean pulmonary arterial pressure were collected every 1.5 h. Cardiac index (CI) was calculated using standard formulas; the body surface area was estimated from Mitchell’s sheep-specific formula[4].

***Experimental Protocol.***

After the surgical procedures, the animal was allowed to stabilize for 1 h. In all groups, plasmalyte solution and 6% hydroxy-ethyl starch solution (Voluven; Fresenius Kabi, Schelle, Belgium) were titrated to prevent hypovolemia and arterial hypotension.

*Sepsis vs. sham groups.*

In the sepsis group, feces were injected into the abdominal cavity and animals were observed until septic shock (SS) occurred, defined as persisting MAP < 65 mmHg and lactate elevation > 2 mmol/L despite adequate fluid resuscitation[5]; than animals were sacrificed using IV potassium chloride. From our experience with this model, we saw that after septic shock the respiratory parameters are difficult to be controlled without a timing vasopressor therapy; since PaCO_2_ highly influences cerebrovascular resistance and dCA assessment, animals in the sepsis group were sacrificed after septic shock. In the sham group, data were collected for 12.5 h; then animals were sacrificed using IV potassium chloride. All analyses were performed offline, using built-in functions and custom scripts in Matlab (The MathWorks, Natick, MA, USA). Since the interval to develop septic shock differed between septic animals, data from sepsis and sham animals were subdivided into four equal time epochs. The first 30 minutes for each epoch were selected and they represented the first four time points (T1_sepsis_-T4_sepsis_); the fifth time point (T5_sepsis_) corresponds to the last 30 minutes of the whole recording. The same set of analysis was performed for each time point in all animals.

*Septic shock group.*

In the septic shock (SS) group, after the initial stabilization period, feces were injected in the abdominal cavity as in the sepsis group. When the response to fluids resuscitation became insufficient, noradrenaline (NA) was started (initial dose 0.5 µg/kg/min) and adjusted to maintain MAP around 80 mmHg. The response to fluid challenge (250 cc infused in 10 minutes) was deemed insufficient when it resulted in a rise in the CO inferior to 15% of the pre-challenge value[6]. The infusion of NA was maintained and titrated up until the MAP did no longer respond to increase in NA dose and fell below 65 mmHg. Data during NA infusion were subdivided into four time epochs and four time points (T1_SS_-4_SS_) were identified by selecting the first 30-minutes for each epoch; in addition, two 30-minutes time epochs were selected, one immediately before the onset of the NA infusion (T0_SS_) and the second immediately after NA withdrawal (T5_SS_). Of note, since the end of the recording in the sepsis group slightly differed from the beginning of NA infusion in the septic shock animals, data collected before T0_SS_ were discarded for purpose of rigour.

***Data analysis.***

*Data pre-processing.*

Physiologically implausible values (MAP > 250 mmHg or < 0 mmHg; CBFv > 5000 blood perfusion units (BPU) or < 100 BPU; CO > 15 L/min or < 0 L/min) and artifacts were visually removed, prior to further analysis.

*Dynamic cerebral autoregulation (dCA).*

CBFv signals from the 2 hemispheres were averaged, yielding a mean-CBFv (mCBF). For dCA assessment, 2 linear methods, one in the time domain (Lxa) and the other in the time-frequency domain (transfer function analysis, TFA), were employed using recorded spontaneous fluctuations of MAP and mCBF.

For Lxa, MAP and mCBF were further averaged on 10-s consecutive windows without overlap, then a Pearson’s correlation coefficient between 30 samples of the averaged values was calculated, obtaining six Lxa values per time point per animal. Lxa can take any value between -1 and 1. Values close to 1 indicate linear correlation between variables and thus poor autoregulation whereas values closer to 0, or negative, indicate good autoregulation[7]. In contrast to the previously published Lx index[8, 9], obtained with CPP instead of MAP, Lxa index has never been validated in animal or human studies; as a consequence, no cutoff values are available to define autoregulatory failure. On the other hand, a close association was found between the two laser-flow-Doppler derived methods, Lx and Lxa, and it has been previously shown in our model[1] that CPP variations are superimposable to MAP ones since intra-cranial pressure do not present notable changes.

TFA was calculated using the Matlab TFA function provided by the Cerebral Autoregulation Research Network[10]. The TFA function allows the estimation of two parameters describing dCA, the gain and the phase shift which reflect, respectively, the relative amplitude and time relationship between the changes in MAP and CBF over a specified frequency range[11]. In case of intact dCA, the gain will be close to zero because MAP oscillations are efficaciously damped, whereas phase shift will be positive, because variations in CBF recover faster than those in MAP, creating a time delay between the oscillations of the two variables which is, by convention, positive[12]. Conversely, in case of loss of dCA, gain will increase and phase shift will approach zero. The statistical reliability of these dCA estimations is based on the magnitude-squared coherence (MSC) function which tests the linear correlation between MAP and CBF[11]. Using Welch periodogram approach, MSC function which is defined as

MSC (*f*) = |S_xy_(*f*)|^2^ / [(S_xx_(*f*) S_yy_(*f*)]

where S_xx_(*f*) is the autospectrum of changes in the signal of the electroencephalogram (EEG), S_yy_(*f*) is the autospectrum of changes in CBF and S_xy_(*f*) is the cross-spectrum between the two signals[11, 13]. MSC can vary between 0, indicating a lack of correlation, and 1. One value of gain and one of phase per time point per animal were obtained. Since in the very low frequency range (VLF, 0.02 < Hz < 0.07), magnitude of spontaneous oscillation in MAP and CBF is small[14] and coherence between these oscillations is generally low[15], most studies excluded VLF data from analysis; instead, as dCA acts as a ‘high-pass filter’, its effect was expected to be even more efficient in the VLF range[11, 16]. Moreover, in a human study in healthy individuals, gain and phase estimates for VLF that were obtained with low coherence in spontaneous oscillations were comparable to those obtained with higher experimentally induced coherence[14], meaning that, in the appropriate settings, VLF give useful and reliable information about dCA. Thus, we plotted only gain and phase in that range of frequencies. Parameters for TFA were window length of 102.4 s, with 59.9% overlap and a Hanning window that led to 41 windows and a spectral resolution of 0.009 Hz. Also, each window contained at least one full period oscillation of the lower frequency considered (0.02 Hz).

*Neurovascular coupling*

The methodological steps are summarized in **Figure 1 [insert Figure 1]**. CBFv from both hemispheres was filtered using a low-pass zero-phase fourth-order Butterworth ﬁlter with a cut-off frequency of 0.25 Hz in order to limit the higher frequency hemodynamics fluctuations (i.e. due to breathing) [17]. The high gamma frequency band was extracted from the ECOG signal using wavelet transform spectral density estimate (*cwt* function in Matlab) and its inverse function (*icwt* function in Matlab) and its envelope (Eγ) was determined (*envelope* function in Matlab). We used the high gamma frequency band as it has been shown to be best reflect neuronal cortical activity driving NVC[18]. Neurovascular coupling was subsequently measured using two different approaches. First, NVC driven by spikes of cortical activity was assessed. Envelope peaks which exceeded one standard deviation above the mean Eγ were considered of a magnitude enough to induce subsequent CBF hemodynamic fluctuations[19] and were detected using the *findpeaks* function in Matlab. For each detected Eγ activity peak, CBFv epochs spanning from 5 s before to 15 s after the peak were selected, detrended (*detrend* function in Matlab) and normalized using the mean and standard deviation calculated on the 5s pre-Eγ peak epoch. If CBFv peak was identified following the Eγ peak (*findpeaks* function in Matlab), the CBF epoch was included for further analysis. For each included CBFv peak, the normalized amplitude and lag from the corresponding Eγ peak were calculated. Since the number of CBFv peaks considered differed between animals, amplitude and lag values were averaged within each animal and only the mean values per animal were used for further analysis to avoid bias from unequal contribution of the different animals. Since Eγ peaks are relatively rare (approximately 1/min), and in order to study the NVC in a broader time scale, we successively assessed the spectral coherence between the ECOG and the CBFv. Cortical neuronal activity presents spontaneous periodic oscillations[20] that are temporally correlated between functionally related areas[21]. These oscillations are accompanied by time-locked changes in blood volume, flow and oxygenation[17, 22]. The coherence between these oscillations has been used as a quantitative measure of NVC in clinical settings[24]. The spectral coherence studies the linear correlation between the spectra of two signals in different frequency bands and it takes the value of one in case of perfect synchrony between signals and zero in case of complete asynchrony[24]. For each time point described above, the spontaneous fluctuations of Eγ (second-level spectrogram) and CBFv were computed with Welch’s periodogram, with an epoch length of 30 minutes, a window length 180 s, and a window overlap 90%. Previous studies reported that the main peaks of the alpha and theta EEG second-level spectrum were located between 0.01 to 0.02 Hz, 0.05 to 0.07 Hz, and 0.1 to 0.25 Hz[20, 25, 26]. Since gamma band oscillates with a similar periodicity[27], these frequency bands were used for ECOG second-level spectrum analysis. For CBFv fluctuations, the following frequency subcomponents where used, according to the literature: from 0.02 to 0.04 and from 0.04 to 0.15[28, 29]. The coherence between Eγ signal and the filtered, detrended and normalized CBFv was then measured by MSC, using the same parameters as for the second-level periodogram. Since the literature does not provide any, we identified peaks in the MSC spectrum of early sham animals (**Supplemental Figure S1**), and identified their frequency boundaries (0.03 to 0.06 Hz, and 0.06 to 0.13 Hz) and used them for further analysis.

*Alpha-delta ratio*

For each time point described above, ECOG spectrograms were calculated using the Welch’s method. The alpha-delta ratio (ADR) was further calculated, as the ratio between power in the alpha (4-8 Hz) and delta (0.5-4 Hz) frequency bands.

***Statistical Analyses.***

Statistical analyses were performed using Matlab (The MathWorks, Natick, MA, USA). A p-value < 0.05 was considered statistically significant. The sepsis group was compared to the sham group. In the septic shock group, each animal served as its own control; analysis were performed between time points during NA infusion. While NA is known to affect dCA, this effect is lost in case of sepsis[30]. Consequently, it would have been misleading to compare septic animals under NA infusion with sham animals without vasopressors. This also allowed reducing the number of animals.

The Kolmogorov-Smirnov test was performed to assess the normal distribution of values. Data are presented as median and interquartile range (IQR) or median and median absolute deviation. Wilcoxon signed rank test and Friedman test were used to analyze variables differences in time within single groups, Wilcoxon rank sum test and two-way repeated-measure ANOVA for differences between groups, as appropriate; the linear step-up procedure introduced by Benjamini and Hochberg was applied for controlling the false discovery rate[31]. Tukey-Kramer and Holm post-hoc analysis were employed in case of a corrected p-value < 0.05.

Since a low MSC could be the result of a noisy signal[32], a cut-off value is necessary to avoid unreliable estimations of gain and phase using the TFA method[15]. The MSC thresholds for a specific frequency were calculated using a Monte Carlo simulation (95% confidence interval based on 100 repetitions of MSC estimation of randomly values adopting standardized parameters recommended in[15] and specified before). In case of non-significant coherence, corresponding gain and phase values were excluded from analysis. Similarly, the coherence between Eγ and CBFv in the assessment of NVC was considered statistically significant if its value for a specific frequency was greater than 95% confidence limit calculated by a Monte Carlo simulation.

**REFERENCES**

1. Taccone FS, Su F, Pierrakos C, He X, James S, Dewitte O, Vincent J-L, Backer DD (2010) Cerebral microcirculation is impaired during sepsis: an experimental study. Crit Care 14:1–10 . https://doi.org/10.1186/cc9205

2. Taccone FS, Su F, Deyne CD, Abdellhai A, Pierrakos C, He X, Donadello K, Dewitte O, Vincent J-L, Backer DD (2014) Sepsis Is Associated With Altered Cerebral Microcirculation and Tissue Hypoxia in Experimental Peritonitis*. Crit Care Med 42:e114 . https://doi.org/10.1097/ccm.0b013e3182a641b8

3. Taccone FS, Castanares-Zapatero D, Peres-Bota D, Vincent J-L, Berre’ J, Melot C (2010) Cerebral Autoregulation is Influenced by Carbon Dioxide Levels in Patients with Septic Shock. Neurocrit Care 12:35–42 . https://doi.org/10.1007/s12028-009-9289-6

4. Berman A (2003) Effects of Body Surface Area Estimates on Predicted Energy Requirements and Heat Stress. J Dairy Sci 86:3605–3610 . https://doi.org/10.3168/jds.S0022-0302(03)73966-6

5. Singer M, Deutschman CS, Seymour CW, Shankar-Hari M, Annane D, Bauer M, Bellomo R, Bernard GR, Chiche J-D, Coopersmith CM, Hotchkiss RS, Levy MM, Marshall JC, Martin GS, Opal SM, Rubenfeld GD, van der Poll T, Vincent J-L, Angus DC (2016) The Third International Consensus Definitions for Sepsis and Septic Shock (Sepsis-3). JAMA 315:801 . https://doi.org/10.1001/jama.2016.0287

6. Cecconi M, De Backer D, Antonelli M, Beale R, Bakker J, Hofer C, Jaeschke R, Mebazaa A, Pinsky MR, Teboul JL, Vincent JL, Rhodes A (2014) Consensus on circulatory shock and hemodynamic monitoring. Task force of the European Society of Intensive Care Medicine. Intensive Care Med 40:1795–1815 . https://doi.org/10.1007/s00134-014-3525-z

7. Zeiler FA, Donnelly J, Cardim D, Menon DK, Smielewski P, Czosnyka M (2018) ICP Versus Laser Doppler Cerebrovascular Reactivity Indices to Assess Brain Autoregulatory Capacity. Neurocrit Care 28:194–202 . https://doi.org/10.1007/s12028-017-0472-x

8. Brady KM, Lee JK, Kibler KK, Smielewski P, Czosnyka M, Easley RB, Koehler RC, Shaffner DH (2007) Continuous Time-Domain Analysis of Cerebrovascular Autoregulation Using Near-Infrared Spectroscopy. Stroke 38:2818–2825 . https://doi.org/10.1161/STROKEAHA.107.485706

9. Lam JM, Hsiang JN, Poon WS (1997) Monitoring of autoregulation using laser Doppler flowmetry in patients with head injury. J Neurosurg 86:438–445 . https://doi.org/10.3171/jns.1997.86.3.0438

10. Cerebral Autoregulation Research Network

11. Zhang R, Zuckerman JH, Giller CA, Levine BD (1998) Transfer function analysis of dynamic cerebral autoregulation in humans. Am J Physiol 274:H233–41

12. van Beek AH, Claassen JA, Rikkert MGO, Jansen RW (2008) Cerebral Autoregulation: An Overview of Current Concepts and Methodology with Special Focus on the Elderly. J Cereb Blood Flow Metab 28:1071–1085 . https://doi.org/10.1038/jcbfm.2008.13

13. Govindan RB, Massaro AN, Andescavage NN, Chang T, du Plessis A (2014) Cerebral Pressure Passivity in Newborns with Encephalopathy Undergoing Therapeutic Hypothermia. Front Hum Neurosci 8: . https://doi.org/10.3389/fnhum.2014.00266

14. Claassen JAHR, Levine BD, Zhang R (2009) Dynamic cerebral autoregulation during repeated squat-stand maneuvers. J Appl Physiol 106:153–160 . https://doi.org/10.1152/japplphysiol.90822.2008

15. Claassen JA, Abeelen ASM den, Simpson DM, Panerai RB, (CAet) on behalf of the international CARN (2016) Transfer function analysis of dynamic cerebral autoregulation: A white paper from the International Cerebral Autoregulation Research Network. J Cereb Blood Flow Metab 36:665–680 . https://doi.org/10.1177/0271678x15626425

16. Panerai RB (2007) Cerebral Autoregulation: From Models to Clinical Applications. Cardiovasc Eng 8:42–59 . https://doi.org/10.1007/s10558-007-9044-6

17. Saka (2010) Linear superposition of sensory-evoked and ongoing cortical hemodynamics. Front Neuroenergetics. https://doi.org/10.3389/fnene.2010.00023

18. Bergel A, Deffieux T, Demené C, Tanter M, Cohen I (2018) Local hippocampal fast gamma rhythms precede brain-wide hyperemic patterns during spontaneous rodent REM sleep. Nat Commun 9:5364 . https://doi.org/10.1038/s41467-018-07752-3

19. Bruyns‐Haylett M, Harris S, Boorman L, Zheng Y, Berwick J, Jones M (2013) The resting‐state neurovascular coupling relationship: rapid changes in spontaneous neural activity in the somatosensory cortex are associated with haemodynamic fluctuations that resemble stimulus‐evoked haemodynamics. Eur J Neurosci 38:2902–2916 . https://doi.org/10.1111/ejn.12295

20. Novak P, Lepicovska V, Dostalek C (1992) Periodic amplitude modulation of EEG. Neurosci Lett 136:213–215 . https://doi.org/10.1016/0304-3940(92)90051-8

21. Peltier S (2002) T2* Dependence of Low Frequency Functional Connectivity. NeuroImage 16:985–992 . https://doi.org/10.1006/nimg.2002.1141

22. Kwong KK, Belliveau JW, Chesler DA, Goldberg IE, Weisskoff RM, Poncelet BP, Kennedy DN, Hoppel BE, Cohen MS, Turner R (1992) Dynamic magnetic resonance imaging of human brain activity during primary sensory stimulation. Proc Natl Acad Sci 89:5675–5679 . https://doi.org/10.1073/pnas.89.12.5675

23. Leopold DA (2003) Very Slow Activity Fluctuations in Monkey Visual Cortex: Implications for Functional Brain Imaging. Cereb Cortex 13:422–433 . https://doi.org/10.1093/cercor/13.4.422

24. Govindan RB, Massaro A, Chang T, Vezina G, du Plessis A (2016) A novel technique for quantitative bedside monitoring of neurovascular coupling. J Neurosci Methods 259:135–142 . https://doi.org/10.1016/j.jneumeth.2015.11.025

25. Novak P, Lepicovska V (1992) Slow modulation of EEG: NeuroReport 3:189–192 . https://doi.org/10.1097/00001756-199202000-00017

26. Admiraal MM, Gilmore EJ, Van Putten MJAM, Zaveri HP, Hirsch LJ, Gaspard N (2017) Disruption of Brain–Heart Coupling in Sepsis: J Clin Neurophysiol 34:413–420 . https://doi.org/10.1097/WNP.0000000000000381

27. Colgin LL, Denninger T, Fyhn M, Hafting T, Bonnevie T, Jensen O, Moser M-B, Moser EI (2009) Frequency of gamma oscillations routes flow of information in the hippocampus. Nature 462:353–357 . https://doi.org/10.1038/nature08573

28. Zhang Z, Khatami R (2014) Predominant endothelial vasomotor activity during human sleep: a near-infrared spectroscopy study. Eur J Neurosci 40:3396–3404 . https://doi.org/10.1111/ejn.12702

29. Bosch BM, Bringard A, Ferretti G, Schwartz S, Iglói K (2017) Effect of cerebral vasomotion during physical exercise on associative memory, a near-infrared spectroscopy study. Neurophotonics 4:041404 . https://doi.org/10.1117/1.NPh.4.4.041404

30. Berg RM, Plovsing RR, Bailey DM, Holstein-Rathlou N-H, Møller K (2015) The dynamic cerebral autoregulatory adaptive response to noradrenaline is attenuated during systemic inflammation in humans. Clin Exp Pharmacol Physiol 42:740–746 . https://doi.org/10.1111/1440-1681.12421

31. Yoav Benjamini, Yosef Hochberg (1995) Controlling The False Discovery Rate - A Practical And Powerful Approach To Multiple Testing. Journal of the Royal Statistical Society Series B: Methodological 289–300 . https://doi.org/10.2307/2346101

32. Giller CA (1990) The Frequency-Dependent Behavior of Cerebral Autoregulation: Neurosurgery 27:362–368 . https://doi.org/10.1227/00006123-199009000-00004

**SUPPLEMENTAL FIGURE LEGENDS**

**Supplemental Figure S1.** **Mean of the magnitude-squared coherence (MSC) of the sham group.** Gray areas highlight the frequency bands used for further analysis (0.03-0.06 Hz and 0.06-0.13 Hz) around clearly identifiable peaks at 0.04 and 0.12 Hz.

**Supplemental Figure S2. Temporal evolution of neurovascular coupling. Cerebral blood flow velocity changes in response to spikes of cortical activity.** Percentage of ECOG gamma activity (Eγ) peaks followed by cerebral blood flow velocity (CBFv) peaks (A-B) and amplitude (C,D) and lag of the CBFv peaks (E-F) in sepsis vs. sham (A,C,E) and septic shock (B,D,F). No significant difference was observed. Data are presented as median ± mean absolute deviation.

**Supplemental Figure S3a. Results of comparative analysis** **for frequency of cerebral blood flow velocity (CBFv) spectral power (A-D) with time**. Evolution in time of CBFv spectral power in the frequency bands 0.02-0.04 Hz (A-B) and 0.04-0.15 Hz (C-D), in sepsis vs. sham (A,C) and septic shock (B,D), expressed as median and inter-quartile range. No differences were reported between sham and sepsis animals for CBFv (frequency band 0.02-0.04 Hz, p = 0.07; 0.04-0.15 Hz, p = 0.22) by two ways RMANOVA test analysis. In the septic shock group, the CBFv power at T1_SS_ (first time point immediately after noradrenaline administration) was statistically higher than that at T4_SS_ (last time point prior to cessation of noradrenaline) for both considered frequency bands (0.02-0.04 Hz, p = 0.049; 0.04-0.15 Hz, p = 0.008). *, p < 0.05.

**Supplemental Figure S3b. Results of comparative analysis** **for frequency of ECOG gamma activity (Eγ) second-level spectral power (A-F) with time**. Evolution in time of Eγ second-level spectral power in the frequency bands 0.01-0.02 Hz (A-B), 0.05-0.07 Hz (C-D), 0.1-0.25 (E-F), in sepsis vs. sham (A,C,E) and septic shock (B,D,F), expressed as median and inter-quartile range. No differences were reported neither between sham and sepsis animals (frequency band 0.01-0.02 Hz, p = 0.15; 0.05-0.07 Hz, p = 0.14; 0.1-0.25 Hz, p = 0.24) nor in the septic shock group (frequency band 0.01-0.02 Hz, p = 0.31; 0.05-0.07 Hz, p = 0.48; 0.1-0.25 Hz, p = 0.86).

**Supplemental Figure S3c. Results of comparative analysis** **for frequency of magnitude-squared coherence (MSC) (A-D) with time**. Evolution in time of MSC between cerebral blood flow velocity (CBFv) and ECOG gamma activity (Eγ) in the frequency bands 0.03-0.06 Hz (A-B) and 0.06-0.13 Hz (C-D), in sepsis vs. sham (A,C) and septic shock (B,D), expressed as median and inter-quartile range. No differences were reported between sham and sepsis animals for coherence (0.03-0.06 Hz, p = 0.42; 0.06-0.13 Hz, p = 0.42) by two ways RMANOVA test analysis. In the septic shock group, the EEG-CBF coherence at T1_SS_ (first time point immediately after noradrenaline administration) was statistically different than that at T4_SS_ (last time point prior to cessation of noradrenaline) only for 0.03-0.06 Hz frequency band (p < 0.001; 0.06-0.15 Hz, p = 0.07). **, p < 0.01.
