# Supplementary figures and images for: Cerebral autoregulation and neurovascular coupling are progressively impaired during septic shock: an experimental study
Source: Intensive Care Med Exp. 2020 Aug 14;8:44. doi: 10.1186/s40635-020-00332-0 (PMC7426896; doi:10.1186/s40635-020-00332-0)

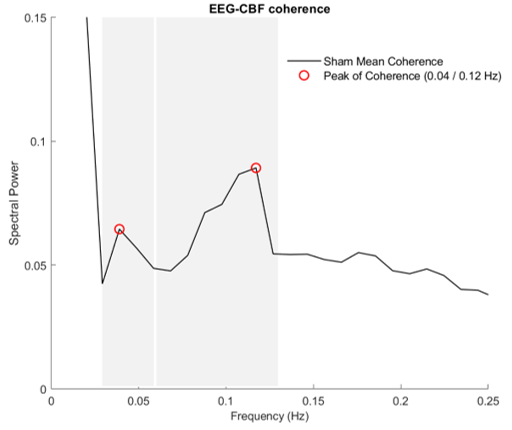

Supplement: Supplementary file 1 — Additional file 1. This section contains five additional figures and a word file with detailed Materials and Methods section and figure legends. Supplemental figure S1 provides a representation of power spectra of magnitude-squared coherence in sham group used in the neurovascular coupling analysis process, while supplemental figure S2 and S3 (a,b,c) provide additional results pertaining to neurovascular coupling analysis. [file 40635_2020_332_MOESM1_ESM.zip › supplemental figure S1.png]

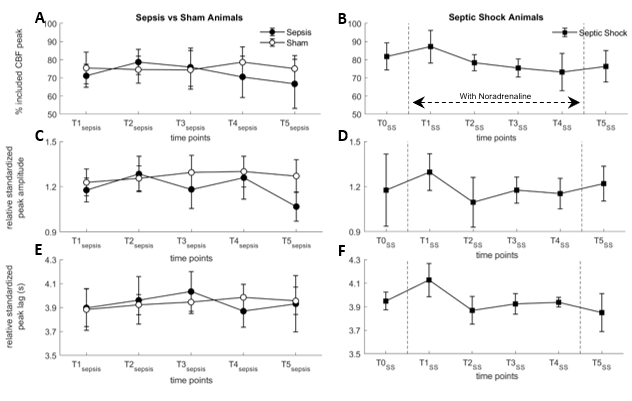

Supplement: Supplementary file 1 — Additional file 1. This section contains five additional figures and a word file with detailed Materials and Methods section and figure legends. Supplemental figure S1 provides a representation of power spectra of magnitude-squared coherence in sham group used in the neurovascular coupling analysis process, while supplemental figure S2 and S3 (a,b,c) provide additional results pertaining to neurovascular coupling analysis. [file 40635_2020_332_MOESM1_ESM.zip › supplemental figure S2.png]

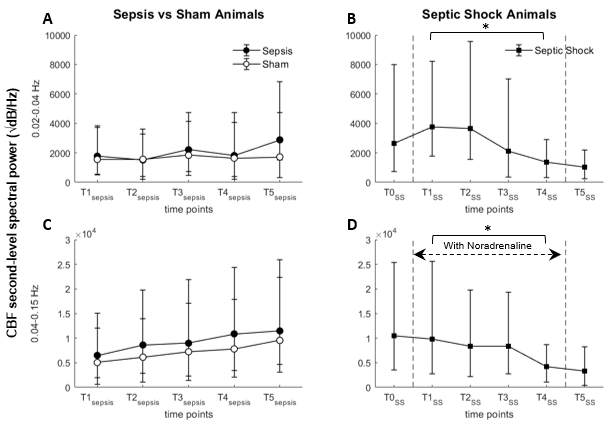

Supplement: Supplementary file 1 — Additional file 1. This section contains five additional figures and a word file with detailed Materials and Methods section and figure legends. Supplemental figure S1 provides a representation of power spectra of magnitude-squared coherence in sham group used in the neurovascular coupling analysis process, while supplemental figure S2 and S3 (a,b,c) provide additional results pertaining to neurovascular coupling analysis. [file 40635_2020_332_MOESM1_ESM.zip › supplemental figure S3a.png]

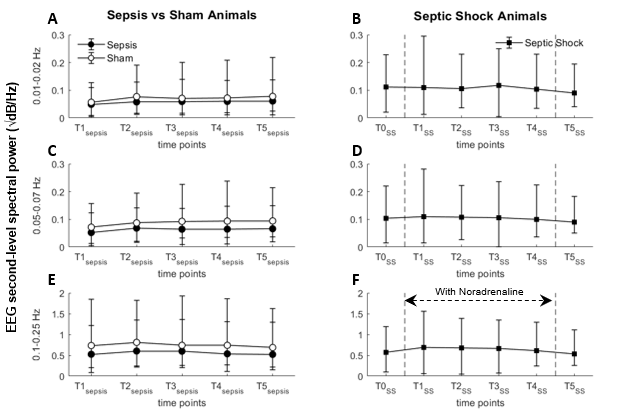

Supplement: Supplementary file 1 — Additional file 1. This section contains five additional figures and a word file with detailed Materials and Methods section and figure legends. Supplemental figure S1 provides a representation of power spectra of magnitude-squared coherence in sham group used in the neurovascular coupling analysis process, while supplemental figure S2 and S3 (a,b,c) provide additional results pertaining to neurovascular coupling analysis. [file 40635_2020_332_MOESM1_ESM.zip › supplemental figure S3b.png]

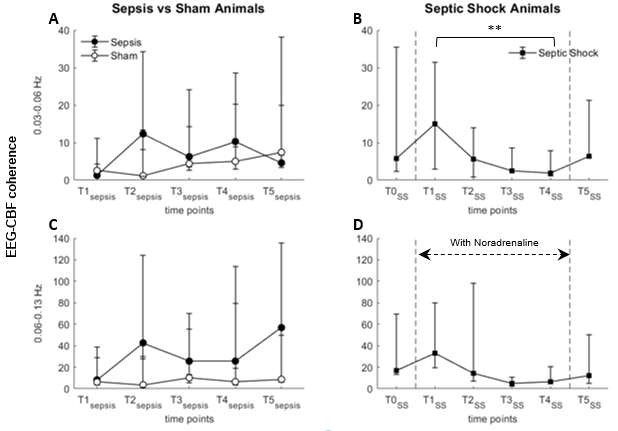

Supplement: Supplementary file 1 — Additional file 1. This section contains five additional figures and a word file with detailed Materials and Methods section and figure legends. Supplemental figure S1 provides a representation of power spectra of magnitude-squared coherence in sham group used in the neurovascular coupling analysis process, while supplemental figure S2 and S3 (a,b,c) provide additional results pertaining to neurovascular coupling analysis. [file 40635_2020_332_MOESM1_ESM.zip › supplemental figure S3c.png]
